# Epigenome-Wide Association Study of Prostate Cancer in African Americans Identifies DNA Methylation Biomarkers for Aggressive Disease

**DOI:** 10.3390/biom11121826

**Published:** 2021-12-03

**Authors:** Yifan Xu, Chia-Wen Tsai, Wen-Shin Chang, Yuyan Han, Maosheng Huang, Curtis A. Pettaway, Da-Tian Bau, Jian Gu

**Affiliations:** 1 Department of Epidemiology, The University of Texas MD Anderson Cancer Center, Houston, TX 77030, USA; yxu13@mdanderson.org (Y.X.); wenwen816@gmail.com (C.-W.T.); halittlemelon@hotmail.com (W.-S.C.); mshuang@mdanderson.org (M.H.); 2 Terry Fox Cancer Research Laboratory, China Medical University Hospital, Taichung 404332, Taiwan; artbau2@gmail.com; 3 School of Biological Sciences, University of Northern Colorado, Greeley, CO 80639, USA; yuyan.han@unco.edu; 4 Department of Urology, The University of Texas MD Anderson Cancer Center, Houston, TX 77030, USA; cpettawa@mdanderson.org; 5 Department of Bioinformatics and Medical Engineering, Asia University, Taichung 41354, Taiwan

**Keywords:** prostate cancer, African American, aggressive disease, DNA methylation, leukocytes

## Abstract

DNA methylation plays important roles in prostate cancer (PCa) development and progression. African American men have higher incidence and mortality rates of PCa than other racial groups in U.S. The goal of this study was to identify differentially methylated CpG sites and genes between clinically defined aggressive and nonaggressive PCa in African Americans. We performed genome-wide DNA methylation profiling in leukocyte DNA from 280 African American PCa patients using Illumina MethylationEPIC array that contains about 860K CpG sties. There was a slight increase of overall methylation level (mean β value) with the increasing Gleason scores (GS = 6, GS = 7, GS ≥ 8, P for trend = 0.002). There were 78 differentially methylated CpG sites with P < 10^−4^ and 9 sites with P < 10^−5^ in the trend test. We also found 77 differentially methylated regions/genes (DMRs), including 10 homeobox genes and six zinc finger protein genes. A gene ontology (GO) molecular pathway enrichment analysis of these 77 DMRs found that the main enriched pathway was DNA-binding transcriptional factor activity. A few representative DMRs include HOXD8, SOX11, ZNF-471, and ZNF-577. Our study suggests that leukocyte DNA methylation may be valuable biomarkers for aggressive PCa and the identified differentially methylated genes provide biological insights into the modulation of immune response by aggressive PCa.

## 1. Introduction

Prostate cancer (PCa) is the most common cancer and second leading cause of cancer death in American men, with an estimated 248,530 new cases and 34,130 deaths from PCa in the U.S. in 2021 [1]. African American men have the highest incidence and mortality rates among all the racial/ethnic groups in the U.S. [2,3]. Prostate-specific antigen (PSA) testing has enabled the detection of PCa at early stages and greatly improved the survival of PCa. The majority of PSA screening-detected PCa are localized, indolent, and not life-threatening. However, most of the PCa patients opt to receive upfront aggressive therapies (radical proctectomy and radiotherapy) that are often associated with significant morbidity, thus overdiagnosis and overtreatment for localized PCa patients have become a major clinical problem. The major reason for the overtreatment is that is the current prognostic algorithms are predominantly composed of clinical variables, such as PSA at diagnosis, Gleason Score (GS), and tumor stage, which are not accurate enough to distinguish aggressive from nonaggressive diseases at the time of disease presentation. Patients with the same clinical features, for example, those with the same GS of 7, often have completely different prognoses—some are dormant and some progress to metastatic disease. Independent biomarkers may be able to supplement clinical variables to predict the clinical course of PCa patients and improve risk stratification of PCa patients for better-informed clinical management.

PCa tumors, particularly localized tumors, have fewer genetic mutations than other adult solid tumors [4,5]. In a large-scale whole genome and whole exome sequence analysis of nearly 500 localized PCa tumors (GS ≤ 7), no gene was mutated in more than 10% of tumors, and only six genes were mutated in more than 2% of tumors [6]. The mutation frequencies in African American PCa patients are the lowest among all the different racial groups [7]. These somatic mutations are not associated with PCa aggressiveness. Epigenetic changes, including DNA methylation, on the other hand, play prominent roles in PCa development and progression, and may serve as promising biomarkers for PCa diagnosis and prognosis [8,9]. A commercially available test, ConfirmMDx, which measures CpG island methylation of GSTP1, RASSF1, and APC in histopathologically negative prostate core biopsies, has been used clinically to predict PCa and high-grade PCa in repeat biopsies [10,11,12]. In addition to DNA methylation in tumor tissues, DNA methylation in peripheral blood leukocytes has also attracted great interest as a predictor of cancer risks and outcomes [13,14,15,16,17,18,19,20,21,22,23]. Recent studies have applied an epigenome-wide association study (EWAS) approach using Illumina’s high-density methylation arrays to identify specific CpG sites in leukocyte DNA that are differentially methylated between European ancestry PCa cases and controls, as well as between low-grade and high-grade PCa patients [21,22,23]. However, no study has performed whole epigenome-wide profiling of CpG site methylations in leukocytes of African American PCa patients.

In this study, we performed an EWAS of African American PCa patients and identified specific leukocyte CpG site methylations that may serve as biomarkers for the aggressive PCa in African Americans.

## 2. Materials and Methods

### 2.1. Study Population

This study included 280 self-reported African American men with histologically confirmed prostate adenocarcinoma. All patients were treated and followed up at the University of Texas MD Anderson Cancer Center. Blood specimens were collected at the time of diagnosis, before treatments. Clinical and follow-up data, which included date of diagnosis, performance status, clinical stage, Gleason scores, PSA levels at diagnosis and follow-up, and treatment (e.g., prostatectomy, radiotherapy, and hormone therapy), were extracted from electronic medical records. The study was approved by the Institutional Review Board of MD Anderson Cancer Center. All patients signed an informed consent form.

### 2.2. Whole Genome Methylation Profiling Using Illumina Human MethylationEPIC Beadchip

The whole genome CpG site methylation profiling was performed using Illumina human MethylationEPIC Beadchip, as previously described [22,23]. The MethylationEPIC Beadchip contains over 850,000 CpG sites across the human genome, among which 54% are located within gene promoters, 30% in gene bodies, and 16% in intergenic regions (16%) [24]. The CpG sites in CpG island are enriched on the MethylationEPIC chip, accounting for 19% of all the CpG sites on the chip [24]. Briefly, 500 ng genomic DNA from peripheral blood was treated with sodium bisulfite using the EZ DNA Methylation-Gold Kit (Zymo Research, Irvine, CA) following manufacturer’s protocol. Bisulfite-treated DNA was hybridized to the MethylationEPIC chip according to the manufacturer’s protocol. Beadchips were scanned on an Illunima HiScan SQ. The fluorescence intensities of the images were extracted using Genome Studio Methylation Module.

### 2.3. Bioinformatics and Data Analyses

Raw fluorescence intensity data (idat files) were processed and normalized using the minfi R package [25]. The methylation status of each CpG site was shown as β-value, calculated as the ratio of the fluorescence intensity signals of the methylated (M) and unmethylated (U) alleles [25]. β values range between 0 (non-methylated) and 1 (completely methylated). The batch effect was removed using the ComBat function from R package [26]. The variation in peripheral blood white cell proportions was controlled for using cell proportion estimates generated by the estimateCellCounts function in minfi R package [27]. The differentially methylated CpG sites (DMCs) were identified by comparing the β values of each CpG site between GS = 6, GS = 7, and GS ≥ 8 groups using a trend test. To determine the prognostic value of each DMC, we dichotomized patients into high- and low-methylation groups based on the median β value and used the low-methylation group as the reference group. A multivariable Cox proportional hazards model was used to estimate the hazard ratio (HR) and 95% confidence interval (CI) for the associations of each DMC and biochemical recurrence (BCR), adjusting for age, PSA level, Gleason score, clinical stage, and treatment. We also identified differentially methylated regions/genes (DMRs). We set the criteria of DMR as having at least 7 consecutive CpG sites and the largest distance between each CpG site as 500 base pairs.

## 3. Results

### 3.1. Patient Characteristics

We performed methylation profiling in leukocyte DNA from 280 African American PCa patients. Table 1 shows the selected patient characteristics. The median age of patients was 58 (range, 39–83) years. The majority (57.9%) were never smokers, whereas 12.1% were current smokers and 30% former smokers. Nearly half (49.1%) of patients were obese (body mass index [BMI] ≥ 30) and another 36.8% were overweight (25 ≤ BMI <30). There were 82 (29.3%) GS = 6, 162 (58.8%) GS = 7, and 36 (12.9%) GS ≥ 8 patients. The patients were predominantly stage I (57.9%) and stage II (36.1%) patients and had PSA < 10 ng/mL (77.2%).

### 3.2. Leukocyte CpG Methylation Pattern in African American PCa Patients

**Figure 1:**
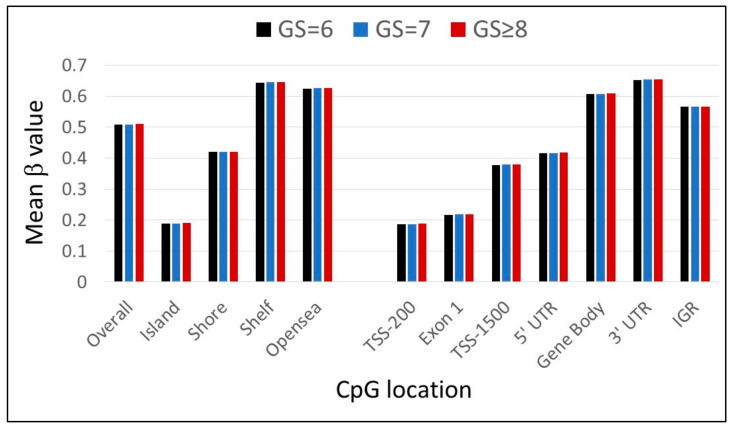
Comparisons of mean β values of CpG sites by locations of CpG sites relative to islands and gene structure to show the overall leukocyte DNA methylation in African American prostate cancer patients with different Gleason scores. Abbreviations: TSS200: within 200 bp of the transcription start site (TSS); TSS1500: within 1500 bp of the TSS; UTR: untranslated region; IGR: intergenic regions.

We first compared the genome-wide CpG methylation levels between patients with different Gleason scores. Overall, the mean methylation levels of all the assayed 860K CpG sites were slightly higher in GS ≥ 8 (mean β = 0.5091) and GS = 7 (mean β = 0.5088) than in GS = 6 (mean β = 0.5076) patients (P for trend = 0.002). As expected, the mean methylation levels were dramatically different depending on the locations of CpG sites, with CpG sites within or near the transcriptionally active regions showing the lowest methylation levels (Figure 1). CpG islands play critical roles in regulating gene expression and mostly have very low methylation (mean β = 0.189), allowing active transcription, while CpG sites outside of CpG islands have much higher methylations (mean β = 0.420, 0.645, and 0.625 for CpG sites in shores, shelfs, and open seas relative to islands). When CpG sites were grouped by locations relative to genes, the mean β values showed a progressive increase associated with the increased distance to the core promoter. CpG sites within 200 base pairs of the transcription start site (TSS200) had the lowest methylation (mean β = 0.187), followed by those in the Exon 1 (mean β = 0.218), TSS-1500 (within 1500 base pairs of TSS) (mean β = 0.378), 5′ untranslated region (5′-UTR) (mean β = 0.417), intergenic region (IGR) (mean β = 0.566), gene body (mean β = 0.608), and 3′-UTR (mean β = 0.653) (Figure 1).

We then performed a trend test to identify individual CpG sites that showed significant trends of increasing or decreasing methylation associated with increasing Gleason scores. There were 52,456 differentially methylated CpG sites with a nominal significance (P < 0.05), 10,734 CpG sites with P < 0.01, 993 sites with P < 0.001, 78 sites with P < 10^−4^, and 9 sites with P < 10^−5^. About 80% of these DMCs showed a progressive increase of methylation with increasing GS (Table 2).

To test the prognostic value of these DMCs, we used a multivariable Cox proportional hazards model to determine the associations of these DMCs with biochemical recurrence (BCR). Among these top DMCs, only two were independently associated with BCR at a significance level of 0.05 (Table 2). High methylation at cg16432885 and cg00915676 was associated with significantly increased risks of BCR (HR = 3.66, 95% CI, 1.33–10.11, P = 0.012 and 3.24, 95% CI, 1.12–9.4, P = 0.030, respectively). The association of high methylation with worse prognosis is consistent with increased methylation level in patients with higher Gleason score at these two CpG sites.

### 3.3. Differentially Methylated Regions/Genes (DMRs) in High-Grade African American PCa Patients

**Figure 2:**
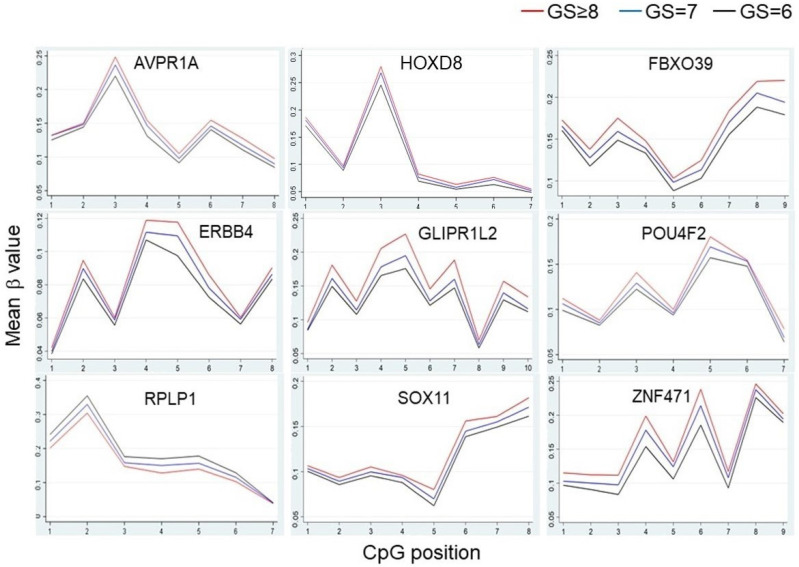
Representative differentially methylated genes between African American prostate cancer patients with high and low Gleason scores.

Due to the fact that DMRs are more likely to be functionally important than scattered individual DMCs, we next searched for DMRs in which at least seven CpGs showed consistently increased or decreased methylation associated with increasing Gleason scores. A total of 77 DMRs were found (Table 3 and Supplemental Table S1). There were 10 homeobox genes (ALX1, HOXC11, HOXD1, HOXD8, HOXD11, LHX8, MSX2, NKX6-2, PAX7, POU4F2) and six zinc finger protein genes (ZBTB16, ZNF83, ZNF471, ZNF577, ZNF714, ZSCAN1). Figure 2 shows the representative DMRs. The majority of these DMRs were found in gene promoter regions with higher methylation levels in patients with higher Gleason scores. We performed a gene ontology (GO) molecular pathway enrichment analysis of these 77 DMRs. The main enriched pathway was DNA-binding transcriptional factor activity, consistent with the major functional roles of homeobox proteins and zinc finger proteins as transcriptional factors.

## 4. Discussion

In this study, we performed genome-wide CpG methylation profiling of leukocyte DNA from 280 African American PCa patients with different Gleason scores to identify intrinsic methylation differences that may serve as predictors of aggressive PCa in African Americans. To our knowledge, this is the first EWAS of PCa in African Americans.

As expected, the mean methylation level was the lowest in the core promoter region (TSS-200) (mean β value < 0.2) and also remained at low levels in Exon 1, TSS-1500, and 5′ UTR (mean β values range between 0.2 and 0.4), but much higher levels were found in the gene body, 3′ UTR, and IGR (mean β values range between 0.5 and 0.7), consistent with the literature of lower promoter methylation allowing a more open chromatin structure and active transcription [28]. When we compared the overall leukocyte methylation levels of GS = 6, GS = 7, and GS ≥ 8 patients, there was a slight increase of methylation level with the increase of Gleason scores. We identified a panel of promising DMCs that are differentially methylated between different Gleason scores. These CpG site methylations may serve as biomarkers for aggressive cancer. More importantly, we identified at least 77 DMRs associated with high-grade PCa. The majority of these DMRs exhibited increased methylation with increased Gleason scores and are located in the promoter island regions of functionally important genes, indicating an overall downregulation of gene expression in leukocytes of high-grade PCa patients, likely affecting immune response, DNA repair, etc., as well as contributing to the aggressive phenotypes.

Among the DMRs between PCa patients with high and low Gleason scores, there were 10 homeobox genes and six zinc finger genes. At least 235 homeobox genes have been identified in the human genome [29]. Homeobox genes contain a highly conserved DNA sequence of about 180 bp encoding the homeodomain of 60 amino acids. Homeodomain proteins act as transcription factors that specifically bind to DNA motifs and regulate the expression of numerous target genes involved in cell proliferation, apoptosis, adhesion, angiogenesis, and DNA repair [30,31]. Homeobox genes are frequently dysregulated in hematological malignancies and solid tumors [31,32]. In addition, homeodomain proteins also play important roles in regulating inflammation and immune response [33,34,35]. Aberrant DNA methylation is one of the major causes of homeobox gene dysregulation during cancer development and progression [36]. Many homeobox genes are hypermethylated in various cancers, including prostate cancer [36]. Two of the identified homeobox genes in our current study, HODX1 and HOXD8, have been shown to play a tumor-suppressor function in various cancers [37,38,39]. Increased methylation of HOXD8 was observed in lymphoma patients compared to normal B cells [40]. Hypermethylation of HOXD8 in urine was associated with disease progression in PCa patients on active surveillance [41]. HOXD8 and several other homeobox genes were hypermethylated in aged muscle tissue compared with young tissue [42]. These previous reports are consistent with our observations of increased HOXD8 methylation in high Gleason score PCa patients. Increased methylation of homeobox genes in leukocyte DNA of PCa patients with high Gleason scores may indicate weakened immune response and suboptimal DNA repair capacity.

The zinc finger proteins are the largest family of transcriptional factors, which function through the binding of the zinc finger domain to specific DNA sequences [43]. In addition to DNA binding, zinc finger proteins can also bind to RNAs and proteins [44]. Zinc finger proteins play crucial roles in transcriptional and post-transcriptional regulation of immune response [45] and are involved in cancer development and progression [46]. Among the identified zinc finger proteins in our study, ZNF471 functions as a tumor suppressor in several cancers and is frequently hypermethylated in tumor tissues [47,48,49,50,51]. Higher levels of ZNF577 methylation in leukocytes have been associated with obesity [52]. As obesity is associated with aggressive PCa [53], ZNF577 methylation may provide a biological link between obesity and PCa progression. Another study showed higher ZNF577 methylation in T-cells from kidney transplant patients who developed de novo skin cancer than those who did not develop skin cancer [54], suggesting increased methylation of ZNF577 may lead to weakened immune response. The exact molecular mechanisms for the roles of these homeodomain and zinc finger proteins play in aggressive PCa warrant further investigation.

Another interesting DMR in our study is SOX11. SOX proteins are a family of about 20 transcriptional factors that have a conserved high-mobility group (HMG) domain that mediates DNA binding [55]. SOX11 has been shown to act as a tumor suppressor in several cancers, including prostate cancer [56,57]. SOX11 is hypermethylated in prostate tumor tissues and the hypermethylation is associated with aggressive clinical features, including higher PSA and Gleason scores [58]. A previous study showed higher SOX11 methylation in leukocyte DNA from gastric patients than that from controls [59]. Our study is the first to show an increased methylation of SOX11 in leukocyte DNA from aggressive PCa patients.

Previously, we performed a similar study of EWAS in European American (EA) prostate cancer patients using the Illumina 450k methylation arrays [23], which covered about 60% of the CpG sites on the MethylationEPIC arrays in this current study. The overall methylation level (mean β value) is higher in AA than EA patients with the same Gleason scores. There were more hypermethylated than hypomethylated CpG sites in patients with higher Gleason scores compared to those with lower Gleason scores. There was very little overlap among the top DMCs and DMRs, but there was significant enrichment of transcriptional factors among DMRs.

CpG methylation in leukocyte DNA sits at the interface between genetics and environment, with longstanding effects on gene expression, inflammation, and immune response [60]. Leukocyte DNA methylation signatures are excellent biomarkers of age and smoking status in a normal population [61,62,63,64]. We compared methylation patterns in PCa patients with different Gleason scores. The age and smoking status distributions in patients with GS6, GS7, and GS ≥ 8 in our study were very similar. In addition, most of our patients were never smokers. The differences of CpG site methylation were consistent when we performed stratified analyses by age group and smoking status (data not shown). The top DMCs in our study (Table 2) were not among those DMCs found in the aging and smoking methylation signatures [61,62,63,64]. Therefore, the DMCs identified in this current study were not likely due to age, smoking, or other potential confounders. Leukocyte DNA methylation profiles have been used to derive systemic inflammation indices [65,66,67,68] and immune cell lineages [60]. We did not observe significant differences in the immune cell subpopulations between patients with different Gleason scores (data not shown), indicating that the differential methylations were not due to differences in immune cell subtypes, but were intrinsic biological changes associated with disease severity across various immune cell types. Leukocyte DNA methylation levels, therefore, may be valuable biomarkers for aggressive PCa patients. However, it is worth noting that the absolute methylation difference (β value difference) at each CpG site between high-grade and low-grade patients was small (generally <0.05). This small difference of leukocyte DNA methylation level between high-grade and low-grade PCa patients is not surprising because of the high background of normal leukocyte methylation.

There were some limitations of this study. Firstly, this was a single-center study. External validations using independent populations are warranted to confirm the DMCs and DMRs identified from this study. Secondly, we only reported nominal significance values (Table 2). None of the DMCs reached genome-wide significance level (P < 5.9 × 10^−8^) after correcting for multiple testing of 850K markers. This is not surprising as an extremely large sample size is always required for genome-wide association studies (GWASs) and EWASs to reach genome-wide significance level. Future large collaborative efforts are needed to significantly increase the sample size and bring the top DMCs to a genome-wide significance level.

In summary, we performed genome-wide DNA methylation profiling of leukocyte DNA in African American PCa patients. We observed slightly increased overall DNA methylation in high-grade PCa patients compared to low-grade PCa patients. We identified a large panel of differentially methylated CpG sites between patients with different Gleason scores. We found 77 differentially methylated genes between high-grade and low-grade patients, and homeodomain protein and zinc finger protein genes were enriched in DMRs. Our study suggests that leukocyte DNA methylation may be a valuable biomarker for aggressive PCa and the identified DMRs provide biological insights into the modulation of immune response by aggressive PCa.

## Figures and Tables

**Table 1:** Selected patient characteristics.

Variables	N (%)
**Age, median (range)**	58 (39–83)
**Smoking status**	
Never smoker	162 (57.9)
Former smoker	84 (30.0)
Current smoker	34 (12.1)
**Body mass index (BMI)**	
<25	33 (14.1)
25 ≤ BMI < 30	86 (36.8)
≥30	115 (49.1)
**Gleason Score**	
6	82 (29.3)
7	162 (58.8)
≥8	36 (12.9)
**Stage**	
T1	162 (57.9)
T2	101 (36.1)
T3	11 (3.9)
T4	6 (2.1)
**PSA at diagnosis**	
<10 ng/mL	216 (77.2)
≤10 and <20 ng/mL	39 (13.9)
≥20 ng/mL	25 (8.9)

**Table 2:** Top Differentially methylated CpG sites (P < 1 × 10^−4^) between different Gleason scores.

CpG ID	β Value			P for Trend	Chr.	Gene	CpG Location	Cox Analysis for BCR	
	GS = 6	GS = 7	GS ≥ 8					HR (95% CI)	*p* Value
cg11039604	0.926	0.94	0.952	1.17 × 10^−6^	3	N/A	IGR-Open sea	1.16 (0.43–3.09)	0.768
cg15610955	0.843	0.85	0.865	3.32 × 10^−6^	2	GPR39	Body-Open sea	1.1 (0.43–2.8)	0.834
cg01097384	0.313	0.339	0.395	3.42 × 10^−6^	11	N/A	IGR-Island	1.14 (0.45–2.91)	0.782
cg02919721	0.674	0.692	0.712	3.84 × 10^−6^	1	N/A	Open sea	1.51 (0.59–3.84)	0.387
cg10283070	0.803	0.801	0.773	4.46 × 10^−6^	9	OR1B1	TSS1500-Open sea	0.74 (0.31–1.75)	0.488
cg22367191	0.259	0.284	0.3	5.73 × 10^−6^	6	N/A	IGR-Shore	0.74 (0.28–1.91)	0.529
cg18092924	0.809	0.82	0.833	7.27 × 10^−6^	20	DIDO1	5′UTR-Shelf	1.02 (0.42–2.46)	0.968
cg02073492	0.845	0.859	0.866	7.48 × 10^−6^	6	N/A	IGR-Open sea	0.94 (0.38–2.34)	0.901
cg03890037	0.157	0.168	0.197	7.50 × 10^−6^	5	LVRN	TSS200-Island	1.09 (0.45–2.66)	0.853
cg24086405	0.679	0.707	0.728	1.38 × 10^−5^	8	DERL1	TSS1500-Shore	1.22 (0.46–3.29)	0.687
cg14299177	0.035	0.034	0.031	1.55 × 10^−5^	8	MTFR1	5′UTR-Shore	1.1 (0.44–2.7)	0.844
cg02512123	0.825	0.843	0.849	1.61 × 10^−5^	1	ZC3H11A	5′UTR-Shelf	2.05 (0.84–5.01)	0.115
cg03828224	0.036	0.041	0.045	1.75 × 10^−5^	5	PPIC	TSS1500-Island	0.95 (0.37–2.44)	0.910
cg16570133	0.08	0.09	0.098	1.85 × 10^−5^	8	N/A	IGR-Shore	0.97 (0.4–2.32)	0.942
cg19614456	0.855	0.862	0.872	1.99 × 10^−5^	1	GABPB2	5′UTR-Open sea	1.51 (0.63–3.64)	0.360
cg27362302	0.181	0.197	0.209	2.20 × 10^−5^	18	GNAL	Exon 1-Island	1.14 (0.46–2.82)	0.779
cg21811204	0.396	0.429	0.44	2.31 × 10^−5^	5	SHROOM1	Body-Island	1.15 (0.49–2.7)	0.748
cg16432885	0.691	0.71	0.721	2.53 × 10^−5^	3	GSK3B	Body-Open sea	3.66 (1.33–10.11)	0.012
cg00915676	0.651	0.663	0.678	2.95 × 10^−5^	7	N/A	IGR-Open sea	3.24 (1.12–9.4)	0.030
cg10481023	0.753	0.774	0.783	3.01 × 10^−5^	6	GABRR2	Body-Open sea	1.31 (0.54–3.19)	0.546
cg15419054	0.087	0.081	0.073	3.15 × 10^−5^	17	NPEPPS	Body-Shore	0.7 (0.29–1.67)	0.418
cg19078430	0.672	0.689	0.708	3.22 × 10^−5^	17	SSH2	Body-Open sea	0.89 (0.38–2.11)	0.797
cg10183781	0.719	0.733	0.756	3.65 × 10^−5^	18	ATP9B	Body-Open sea	1.27 (0.52–3.11)	0.603
cg20171236	0.464	0.484	0.498	3.68 × 10^−5^	4	N/A	IGR-Open sea	2.37 (0.9–6.26)	0.082
cg07160783	0.666	0.662	0.645	3.83 × 10^−5^	16	N/A	IGR-Open sea	0.99 (0.4–2.46)	0.977
cg02747319	0.306	0.313	0.335	3.84 × 10^−5^	2	N/A	IGR-Shore	2.01 (0.81–5.02)	0.134
cg07665241	0.7	0.723	0.736	3.89 × 10^−5^	X	CXorf36	TSS1500-Open sea	1.06 (0.45–2.47)	0.898
cg12058586	0.123	0.113	0.102	4.23 × 10^−5^	12	N/A	IGR-Open sea	0.39 (0.14–1.1)	0.074
cg05135861	0.426	0.418	0.403	4.29 × 10^−5^	18	DLGAP1	5′UTR-Open sea	0.72 (0.28–1.88)	0.503
cg00157515	0.079	0.087	0.1	4.32 × 10^−5^	2	LOC100132215	TSS1500-Island	2.06 (0.77–5.52)	0.151
cg26470340	0.02	0.023	0.027	4.33 × 10^−5^	10	ARHGAP21	5′UTR-Island	1.02 (0.4–2.61)	0.965
cg11945022	0.576	0.594	0.642	4.51 × 10^−5^	7	DYNC1I1	5′UTR-Open sea	0.62 (0.25–1.55)	0.308
cg18303466	0.753	0.76	0.776	4.57 × 10^−5^	1	SLC9A1	TSS1500-Shore	1.19 (0.5–2.84)	0.694
cg11650926	0.781	0.78	0.764	4.59 × 10^−5^	5	N/A	IGR-Open sea	0.67 (0.28–1.61)	0.370
cg15954675	0.723	0.744	0.771	4.65 × 10^−5^	3	SYNPR	Body-Open sea	3.11 (1.08–8.95)	0.036
cg25595028	0.842	0.852	0.86	4.72 × 10^−5^	11	RNF214	Body-Open sea	1.21 (0.49–2.97)	0.676
cg20981146	0.846	0.856	0.871	5.02 × 10^−5^	1	RSBN1	Body-Open sea	1.02 (0.42–2.5)	0.959
cg19734896	0.825	0.812	0.802	5.12 × 10^−5^	1	ILDR2	Body-Shore	0.55 (0.22–1.36)	0.196
cg12911208	0.744	0.731	0.719	5.13 × 10^−5^	6	RP11-73O6.4	5′UTR-Open sea	0.89 (0.36–2.19)	0.793
cg13928649	0.13	0.138	0.156	5.21 × 10^−5^	9	PRDM12	Body-Island	1.35 (0.51–3.55)	0.546
cg05492453	0.101	0.087	0.082	5.32 × 10^−5^	22	FAM19A5	Body-Open sea	0.83 (0.33–2.1)	0.699
cg04492396	0.073	0.057	0.05	5.32 × 10^−5^	8	N/A	IGR-Open sea	0.59 (0.23–1.51)	0.270
cg00290605	0.024	0.028	0.029	5.33 × 10^−5^	22	CTA-342B11.2	TSS200-Island	0.85 (0.35–2.06)	0.721
cg01382153	0.058	0.063	0.066	5.34 × 10^−5^	2	CCDC108	Exon 1-Island	1.11 (0.44–2.79)	0.822
cg00903099	0.131	0.144	0.15	5.37 × 10^−5^	7	HTR5A	TSS200-Shore	1.14 (0.47–2.78)	0.774
cg18285105	0.631	0.652	0.677	5.37 × 10^−5^	9	RP11-87N24.3	TSS1500-Open sea	1.89 (0.77–4.64)	0.167
cg01049417	0.06	0.064	0.07	5.50 × 10^−5^	3	SCHIP1	Body-Island	1.3 (0.5–3.39)	0.595
cg07744841	0.191	0.208	0.221	5.62 × 10^−5^	3	SLC6A11	TSS1500-Island	3.5 (1.21–10.17)	0.021
cg14530623	0.308	0.326	0.343	5.66 × 10^−5^	2	ITSN2	5′UTR-Open sea	1.7 (0.69–4.22)	0.250
cg25252658	0.623	0.644	0.67	5.67 × 10^−5^	7	FKBP6	Body-Open sea	2.08 (0.83–5.24)	0.120
cg03052956	0.252	0.268	0.306	5.70 × 10^−5^	X	ARHGAP36	TSS1500-Shore	1.45 (0.57–3.68)	0.430
cg01909140	0.836	0.842	0.853	6.07 × 10^−5^	9	C9orf3	Body-Open sea	1.57 (0.64–3.81)	0.324
cg11179997	0.137	0.144	0.152	6.42 × 10^−5^	X	N/A	IGR-Island	0.85 (0.34–2.17)	0.737
cg13453374	0.222	0.236	0.261	6.44 × 10^−5^	3	RP11-649A16.1	3′UTR-Open sea	1.15 (0.48–2.78)	0.753
cg21081034	0.177	0.192	0.203	6.57 × 10^−5^	2	LOC100132215	TSS1500-Island	0.73 (0.27–1.98)	0.532
cg16372976	0.494	0.531	0.57	6.74 × 10^−5^	1	RFWD2	Body-Shelf	1.11 (0.45–2.76)	0.824
cg00088299	0.013	0.016	0.018	6.80 × 10^−5^	4	N/A	IGR-Island	2.37 (0.89–6.32)	0.084
cg25339368	0.809	0.817	0.828	6.92 × 10^−5^	17	TBCD	Body-Shore	1.22 (0.49–3.06)	0.673
cg25215047	0.804	0.807	0.837	6.97 × 10^−5^	13	N/A	IGR-Open sea	1.29 (0.54–3.07)	0.565
cg07629204	0.484	0.507	0.534	7.14 × 10^−5^	10	N/A	IGR-Open sea	2.41 (0.8–7.23)	0.118
cg21377071	0.495	0.521	0.541	7.31 × 10^−5^	15	ADAMTSL3	5′UTR-Shore	1.94 (0.77–4.89)	0.160
cg23099959	0.622	0.643	0.665	7.54 × 10^−5^	15	RP11-66B24.2	5′UTR-Shore	0.6 (0.22–1.61)	0.309
cg14824107	0.73	0.721	0.683	7.58 × 10^−5^	17	TBCD	Body-Shore	0.63 (0.25–1.59)	0.326
cg01993946	0.699	0.688	0.671	8.00 × 10^−5^	10	N/A	IGR-Open sea	0.52 (0.21–1.28)	0.156
cg11875624	0.06	0.084	0.083	8.08 × 10^−5^	X	FGF13	5′UTR-Island	0.93 (0.36–2.41)	0.882
cg19004134	0.02	0.021	0.024	8.12 × 10^−5^	6	VTA1	TSS200-Island	1.58 (0.65–3.82)	0.310
cg03854198	0.087	0.093	0.104	8.14 × 10^−5^	12	NTF3	Exon 1-Island	1.49 (0.53–4.15)	0.448
cg13786089	0.092	0.103	0.117	8.28 × 10^−5^	19	ZFR2	TSS200-Island	1.68 (0.67–4.26)	0.271
cg05917797	0.775	0.782	0.792	8.40 × 10^−5^	14	N/A	IGR-Open sea	1.58 (0.64–3.87)	0.318
cg12574406	0.813	0.822	0.834	8.60 × 10^−5^	8	N/A	IGR-Open sea	1.2 (0.48–3.04)	0.693
cg04855249	0.788	0.782	0.77	8.97 × 10^−5^	15	OCA2	Body-Open sea	0.92 (0.37–2.29)	0.859
cg23409289	0.018	0.02	0.023	9.42 × 10^−5^	5	C5orf56	TSS1500-Island	1.46 (0.59–3.62)	0.410
cg12949141	0.593	0.612	0.635	9.48 × 10^−5^	5	PCBD2	Body-Open sea	1.56 (0.61–3.98)	0.354
cg00170540	0.768	0.785	0.791	9.55 × 10^−5^	16	HSDL1	5′UTR-Open sea	1.18 (0.49–2.79)	0.715
cg22851420	0.22	0.238	0.25	9.58 × 10^−5^	1	HPCAL4	Body-Island	0.67 (0.26–1.68)	0.391
cg04921989	0.07	0.077	0.084	9.84 × 10^−5^	2	N/A	IGR-Island	1.23 (0.51–2.95)	0.651
cg13551368	0.508	0.497	0.484	9.87 × 10^−5^	5	N/A	IGR-Open sea	0.5 (0.19–1.3)	0.154
cg06465285	0.776	0.796	0.815	9.95 × 10^−5^	8	N/A	IGR-Open sea	0.79 (0.32–1.94)	0.608

**Table 3:** Differentially methylated genes between different Gleason scores.

ALX1, ANKHD1-EIF4EBP3, ARHGAP15, ATXN7, AVPR1A, BACH2, C6orf174, CD24, CD247,
CLDN11, CLVS2, CSDAP1, DLX6AS, DNASE1L2, ERBB4, ESRP2, FAM171A2, FAM179A,
FBN2, FBXO39, FOXG1, GABRB2, GALNT13, GJB6, GLIPR1L2, GNAS, GNMT, GRIK2, HERC2,
HOXC11, HOXD1, HOXD11, HOXD8, HPSE2, KHDRBS2, KSR2, LHX8, LRP5, MAPK8IP3,
MIR2277, MSX2, NKAIN3, NKX6-2, PAX7, PCDH10, PEG10, PF4, PHYHIPL, PITX2, POU4F2
PPP2R5E, PRH1, PTPN13, RBM33, RPLP1, RSPO2, SFRP2, SGCE, SHROOM1, SLC6A11,
SOX11, SPATA18, SPTBN1, TRIM2, TWIST1, UPB1, WDR60, WDR8, WNT6, WT1, WWP2,
ZBTB16, ZNF471, ZNF577, ZNF714, ZNF83, ZSCAN1

## Data Availability

The data presented in this study are available on request from the corresponding author. The data are not publicly available due to due to privacy information and ethical consideration.

## Supplementary Materials

The following are available online at https://www.mdpi.com/article/10.3390/biom11121826/s1, Table S1: Differentially methylated regions/genes and their CpG site methylations between different Gleason scores in African American prostate cancer patients.
