# Supplementary material for: Epigenome-Wide Association Study of Prostate Cancer in African Americans Identifies DNA Methylation Biomarkers for Aggressive Disease
Source: Biomolecules. 2021 Dec 3;11(12):1826. doi: 10.3390/biom11121826 (PMC8698937; doi:10.3390/biom11121826)
Supplement: Supplementary file 1 [file biomolecules-11-01826-s001.zip › biomolecules-1435363-supplementary.pdf]

Supplemental Table S1. Differentially methylated regions/genes and their CpG site methylations between different Gleason Scores in African American prostate cancer patients

CpG ID	β value			P for trend	Chromo-some	Gene
	GS=6	GS=7	GS≥8			
cg13916459	0.106	0.120	0.127	0.004	12	ALX1
cg25800765	0.092	0.096	0.100	0.037	12	ALX1
cg04245590	0.103	0.108	0.110	0.026	12	ALX1
cg24214152	0.043	0.044	0.047	0.046	12	ALX1
cg24083469	0.057	0.058	0.060	0.045	12	ALX1
cg14116122	0.071	0.075	0.078	0.020	12	ALX1
cg02409351	0.069	0.072	0.076	0.033	12	ALX1
cg15195990	0.796	0.806	0.820	0.013	5	ANKHD1-EIF4EBP3
cg01023982	0.739	0.746	0.754	0.017	5	ANKHD1-EIF4EBP3
cg12177222	0.690	0.713	0.735	0.001	5	ANKHD1-EIF4EBP3
cg03479012	0.801	0.810	0.812	0.004	5	ANKHD1-EIF4EBP3
cg26911562	0.033	0.034	0.036	0.044	5	ANKHD1-EIF4EBP3
cg17416146	0.041	0.041	0.046	0.021	5	ANKHD1-EIF4EBP3
cg11639849	0.033	0.035	0.046	0.008	5	ANKHD1-EIF4EBP3
cg00078221	0.184	0.193	0.200	0.010	5	ANKHD1-EIF4EBP3
cg21833419	0.718	0.723	0.736	0.024	2	ARHGAP15
cg17669276	0.796	0.812	0.826	0.027	2	ARHGAP15
cg09206588	0.818	0.823	0.826	0.042	2	ARHGAP15
cg25026949	0.818	0.823	0.831	0.007	2	ARHGAP15
cg09462438	0.803	0.810	0.821	0.049	2	ARHGAP15
cg21762808	0.715	0.724	0.755	0.000	2	ARHGAP15
cg17056048	0.825	0.830	0.831	0.049	2	ARHGAP15
cg07409629	0.791	0.805	0.818	0.001	2	ARHGAP15
cg01943987	0.457	0.460	0.491	0.039	2	ARHGAP15
cg10136077	0.699	0.713	0.723	0.034	3	ATXN7
cg03413877	0.590	0.607	0.612	0.038	3	ATXN7
cg06077738	0.808	0.813	0.816	0.020	3	ATXN7
cg18138031	0.741	0.753	0.764	0.043	3	ATXN7
cg04226394	0.023	0.024	0.025	0.031	3	ATXN7
cg00745333	0.786	0.795	0.798	0.032	3	ATXN7
cg19542364	0.787	0.794	0.805	0.014	3	ATXN7
cg16668728	0.125	0.132	0.132	0.026	12	AVPR1A
cg24501701	0.144	0.148	0.150	0.050	12	AVPR1A
cg10906284	0.220	0.237	0.248	0.006	12	AVPR1A
cg19987210	0.131	0.146	0.154	0.048	12	AVPR1A
cg10862431	0.091	0.098	0.105	0.018	12	AVPR1A
cg21164131	0.141	0.146	0.155	0.023	12	AVPR1A
cg13631391	0.110	0.117	0.128	0.007	12	AVPR1A
cg12516059	0.085	0.090	0.098	0.037	12	AVPR1A
cg18410117	0.799	0.806	0.807	0.013	6	BACH2

cg04508839	0.039	0.040	0.041	0.044	6	BACH2
cg12234226	0.442	0.453	0.462	0.022	6	BACH2
cg20768122	0.826	0.832	0.842	0.031	6	BACH2
cg09745430	0.055	0.056	0.060	0.022	6	BACH2
cg10135588	0.015	0.016	0.019	0.002	6	BACH2
cg18965377	0.015	0.017	0.018	0.018	6	BACH2
cg10643578	0.022	0.023	0.026	0.035	6	BACH2
cg13276981	0.791	0.798	0.806	0.035	6	C6orf174
cg01047555	0.244	0.257	0.268	0.013	6	C6orf174
cg13612083	0.113	0.121	0.128	0.008	6	C6orf174
cg12462816	0.137	0.144	0.147	0.008	6	C6orf174
cg16001722	0.109	0.115	0.119	0.014	6	C6orf174
cg13841603	0.123	0.127	0.137	0.007	6	C6orf174
cg04084295	0.073	0.076	0.079	0.016	6	C6orf174
cg14683071	0.437	0.447	0.454	0.017		CD24
cg16227841	0.407	0.418	0.426	0.024		CD24
cg13438027	0.479	0.488	0.491	0.035		CD24
cg16061810	0.483	0.493	0.500	0.047		CD24
cg02351050	0.374	0.386	0.395	0.021		CD24
cg01150227	0.563	0.574	0.581	0.041		CD24
cg12439150	0.421	0.448	0.454	0.031		CD24
cg12389252	0.270	0.272	0.288	0.032	1	CD247
cg21161394	0.341	0.353	0.376	0.007	1	CD247
cg06900257	0.159	0.167	0.171	0.015	1	CD247
cg10375409	0.547	0.554	0.566	0.018	1	CD247
cg18858739	0.604	0.616	0.626	0.014	1	CD247
cg25503136	0.798	0.803	0.812	0.015	1	CD247
cg27122449	0.435	0.454	0.458	0.008	1	CD247
cg26880239	0.217	0.225	0.228	0.032	1	CD247
cg20970810	0.642	0.654	0.663	0.016	1	CD247
cg13879776	0.231	0.241	0.243	0.022	3	CLDN11
cg11145160	0.229	0.239	0.248	0.031	3	CLDN11
cg07434518	0.129	0.133	0.136	0.026	3	CLDN11
cg07137845	0.116	0.120	0.125	0.048	3	CLDN11
cg20449692	0.148	0.161	0.167	0.004	3	CLDN11
cg00894757	0.064	0.067	0.071	0.015	3	CLDN11
cg17078427	0.060	0.062	0.064	0.027	3	CLDN11
cg15676162	0.526	0.532	0.565	0.020	6	CLVS2
cg05757803	0.646	0.665	0.684	0.004	6	CLVS2
cg19268752	0.127	0.136	0.139	0.045	6	CLVS2
cg09946253	0.121	0.136	0.141	0.005	6	CLVS2
cg21727532	0.083	0.086	0.090	0.007	6	CLVS2
cg05009934	0.077	0.081	0.086	0.004	6	CLVS2
cg10182317	0.199	0.223	0.237	0.005	6	CLVS2

cg08194879	0.072	0.080	0.087	0.024	6	CLVS2
cg18210226	0.057	0.064	0.066	0.032	6	CLVS2
cg06301178	0.255	0.255	0.267	0.026	16	CSDAP1
cg08980382	0.527	0.535	0.550	0.043	16	CSDAP1
cg16635314	0.044	0.048	0.053	0.027	16	CSDAP1
cg10222027	0.091	0.100	0.103	0.019	16	CSDAP1
cg03131298	0.027	0.031	0.032	0.035	16	CSDAP1
cg04764012	0.063	0.071	0.076	0.039	16	CSDAP1
cg16446484	0.577	0.603	0.615	0.013	16	CSDAP1
cg07788369	0.161	0.166	0.175	0.042	7	DLX6AS
cg09803262	0.179	0.187	0.191	0.033	7	DLX6AS
cg18502142	0.214	0.224	0.231	0.008	7	DLX6AS
cg21545390	0.173	0.183	0.194	0.003	7	DLX6AS
cg12055395	0.105	0.111	0.119	0.006	7	DLX6AS
cg02530022	0.101	0.101	0.108	0.028	7	DLX6AS
cg20109856	0.268	0.282	0.288	0.025	7	DLX6AS
cg12619509	0.323	0.326	0.338	0.044	16	DNASE1L2
cg27557378	0.088	0.094	0.106	0.028	16	DNASE1L2
cg26305896	0.206	0.214	0.237	0.009	16	DNASE1L2
cg12423667	0.272	0.288	0.312	0.004	16	DNASE1L2
cg26415566	0.286	0.294	0.318	0.002	16	DNASE1L2
cg06235653	0.214	0.222	0.251	0.005	16	DNASE1L2
cg09219877	0.158	0.164	0.172	0.006	16	DNASE1L2
cg21463740	0.038	0.040	0.042	0.019	2	ERBB4
cg08042975	0.084	0.090	0.095	0.010	2	ERBB4
cg07015629	0.056	0.059	0.060	0.040	2	ERBB4
cg00787188	0.107	0.112	0.119	0.029	2	ERBB4
cg19079194	0.098	0.110	0.118	0.007	2	ERBB4
cg08870194	0.073	0.078	0.086	0.011	2	ERBB4
cg06149408	0.056	0.059	0.060	0.029	2	ERBB4
cg00011346	0.084	0.086	0.090	0.033	2	ERBB4
cg07808610	0.446	0.453	0.458	0.043	16	ESRP2
cg06723863	0.090	0.104	0.120	0.007	16	ESRP2
cg04513006	0.136	0.147	0.157	0.036	16	ESRP2
cg04328477	0.190	0.203	0.217	0.031	16	ESRP2
cg21690925	0.149	0.155	0.160	0.025	16	ESRP2
cg08694699	0.106	0.111	0.118	0.016	16	ESRP2
cg01177854	0.136	0.138	0.142	0.035	16	ESRP2
cg19836999	0.328	0.339	0.344	0.017	16	ESRP2
cg08886823	0.257	0.266	0.287	0.027	16	ESRP2
cg15909132	0.471	0.493	0.505	0.036	17	FAM171A2
cg17624802	0.329	0.348	0.355	0.023	17	FAM171A2
cg09014329	0.328	0.347	0.351	0.029	17	FAM171A2
cg23766254	0.448	0.472	0.480	0.015	17	FAM171A2

cg22078805	0.435	0.466	0.469	0.016	17	FAM171A2
cg19935040	0.525	0.547	0.556	0.004	17	FAM171A2
cg13661908	0.506	0.512	0.520	0.013	17	FAM171A2
cg25553279	0.732	0.735	0.743	0.016	2	FAM179A
cg17393424	0.696	0.699	0.705	0.048	2	FAM179A
cg20904657	0.342	0.347	0.364	0.022	2	FAM179A
cg03355123	0.773	0.780	0.785	0.004	2	FAM179A
cg09113310	0.581	0.589	0.611	0.033	2	FAM179A
cg21539397	0.527	0.537	0.547	0.011	2	FAM179A
cg06049354	0.591	0.600	0.608	0.007	2	FAM179A
cg17564775	0.091	0.107	0.112	0.012	5	FBN2
cg12940822	0.126	0.145	0.148	0.034	5	FBN2
cg25084878	0.093	0.101	0.106	0.031	5	FBN2
cg02620875	0.086	0.092	0.095	0.046	5	FBN2
cg25532099	0.066	0.069	0.072	0.046	5	FBN2
cg05686497	0.136	0.150	0.162	0.044	5	FBN2
cg27223047	0.137	0.145	0.151	0.027	5	FBN2
cg23341223	0.160	0.166	0.173	0.009	17	FBXO39
cg23729107	0.118	0.128	0.138	0.007	17	FBXO39
cg00376544	0.149	0.159	0.175	0.015	17	FBXO39
cg11871421	0.133	0.139	0.148	0.004	17	FBXO39
cg06091013	0.088	0.098	0.103	0.009	17	FBXO39
cg08843809	0.103	0.113	0.125	0.009	17	FBXO39
cg02093112	0.156	0.170	0.184	0.004	17	FBXO39
cg07540103	0.188	0.205	0.219	0.004	17	FBXO39
cg02374745	0.179	0.194	0.220	0.002	17	FBXO39
cg03523785	0.120	0.123	0.128	0.022	14	FOXG1
cg10912240	0.159	0.163	0.168	0.029	14	FOXG1
cg18299578	0.105	0.108	0.114	0.006	14	FOXG1
cg04525757	0.129	0.133	0.149	0.025	14	FOXG1
cg19714132	0.077	0.081	0.084	0.019	14	FOXG1
cg02681442	0.112	0.119	0.125	0.026	14	FOXG1
cg08120263	0.127	0.135	0.140	0.021	14	FOXG1
cg02991338	0.128	0.142	0.151	0.008	14	FOXG1
cg27006650	0.087	0.097	0.103	0.006	14	FOXG1
cg17525102	0.089	0.092	0.098	0.025	14	FOXG1
cg10300684	0.083	0.088	0.094	0.011	14	FOXG1
cg16582779	0.163	0.168	0.172	0.035	14	FOXG1
cg12150366	0.491	0.502	0.507	0.043	14	FOXG1
cg01543871	0.522	0.539	0.541	0.042	5	GABRB2
cg21901643	0.219	0.232	0.238	0.018	5	GABRB2
cg19971650	0.046	0.047	0.049	0.041	5	GABRB2
cg01939681	0.046	0.048	0.049	0.041	5	GABRB2
cg11042722	0.134	0.141	0.150	0.009	5	GABRB2

cg06600429	0.170	0.175	0.183	0.011	5	GABRB2
cg04744597	0.039	0.041	0.044	0.011	5	GABRB2
cg00252615	0.137	0.139	0.144	0.047	5	GABRB2
cg21998983	0.074	0.078	0.084	0.005	2	GALNT13
cg14635767	0.071	0.078	0.085	0.002	2	GALNT13
cg12424785	0.059	0.063	0.069	0.023	2	GALNT13
cg26983710	0.102	0.114	0.126	0.005	2	GALNT13
cg07547000	0.057	0.065	0.073	0.009	2	GALNT13
cg14739151	0.107	0.117	0.132	0.013	2	GALNT13
cg12079322	0.087	0.097	0.109	0.050	2	GALNT13
cg10033761	0.515	0.519	0.536	0.008	2	GALNT13
cg14630357	0.052	0.056	0.058	0.001	2	GALNT13
cg07380056	0.558	0.564	0.581	0.038	2	GALNT13
cg21833932	0.787	0.798	0.801	0.016	2	GALNT13
cg18145090	0.729	0.738	0.745	0.025	13	GJB6
cg01700504	0.423	0.434	0.437	0.048	13	GJB6
cg01446515	0.188	0.195	0.199	0.048	13	GJB6
cg20972453	0.304	0.315	0.323	0.036	13	GJB6
cg10395448	0.062	0.063	0.066	0.018	13	GJB6
cg07148716	0.041	0.045	0.050	0.027	13	GJB6
cg00777926	0.829	0.830	0.845	0.023	13	GJB6
cg20291222	0.084	0.086	0.096	0.031	12	GLIPR1L2
cg00108944	0.149	0.161	0.181	0.010	12	GLIPR1L2
cg23588049	0.108	0.115	0.128	0.008	12	GLIPR1L2
cg12351126	0.166	0.178	0.206	0.005	12	GLIPR1L2
cg02415057	0.176	0.195	0.227	0.002	12	GLIPR1L2
cg07311024	0.122	0.128	0.146	0.017	12	GLIPR1L2
cg02071292	0.147	0.160	0.188	0.007	12	GLIPR1L2
cg15942481	0.058	0.063	0.070	0.007	12	GLIPR1L2
cg21272279	0.130	0.140	0.157	0.007	12	GLIPR1L2
cg17050806	0.112	0.116	0.134	0.025	12	GLIPR1L2
cg11996914	0.535	0.541	0.551	0.048	20	GNAS
cg21330323	0.523	0.531	0.538	0.023	20	GNAS
cg14728235	0.582	0.589	0.600	0.008	20	GNAS
cg24975842	0.512	0.519	0.533	0.025	20	GNAS
cg18619398	0.385	0.392	0.412	0.050	20	GNAS
cg26102503	0.376	0.388	0.399	0.008	20	GNAS
cg26711395	0.625	0.631	0.641	0.015	20	GNAS
cg09437522	0.391	0.406	0.415	0.006	20	GNAS
cg04019914	0.551	0.562	0.571	0.018	20	GNAS
cg26767990	0.515	0.523	0.540	0.004	20	GNAS
cg25308079	0.570	0.578	0.590	0.012	20	GNAS
cg15222215	0.550	0.555	0.568	0.026	20	GNAS
cg00267746	0.469	0.471	0.489	0.045	20	GNAS

cg22798925	0.549	0.555	0.567	0.017	20	GNAS
cg22639787	0.658	0.664	0.675	0.004	20	GNAS
cg05960039	0.362	0.368	0.384	0.007	20	GNAS
cg09604333	0.429	0.434	0.447	0.043	20	GNAS
cg02867288	0.810	0.814	0.825	0.008	20	GNAS
cg24815792	0.216	0.223	0.246	0.028	6	GNMT
cg21280719	0.386	0.400	0.423	0.041	6	GNMT
cg10862848	0.363	0.374	0.391	0.029	6	GNMT
cg25671484	0.487	0.498	0.519	0.019	6	GNMT
cg04013093	0.063	0.067	0.071	0.047	6	GNMT
cg19070139	0.047	0.052	0.063	0.048	6	GNMT
cg10056627	0.190	0.197	0.210	0.006	6	GNMT
cg27451362	0.084	0.098	0.103	0.001	6	GRIK2
cg06247406	0.135	0.139	0.142	0.018	6	GRIK2
cg13080565	0.258	0.264	0.279	0.003	6	GRIK2
cg05942459	0.110	0.117	0.126	0.007	6	GRIK2
cg24301620	0.272	0.283	0.294	0.005	6	GRIK2
cg18193094	0.110	0.117	0.120	0.005	6	GRIK2
cg10591607	0.111	0.117	0.118	0.013	6	GRIK2
cg21635870	0.162	0.163	0.171	0.043	6	GRIK2
cg24680758	0.622	0.642	0.643	0.004	6	GRIK2
cg16895086	0.957	0.958	0.960	0.042	15	HERC2
cg20080320	0.625	0.637	0.652	0.009	15	HERC2
cg07945425	0.936	0.936	0.941	0.004	15	HERC2
cg16512924	0.735	0.748	0.758	0.015	15	HERC2
cg19563355	0.793	0.793	0.814	0.043	15	HERC2
cg20524527	0.448	0.459	0.460	0.027	15	HERC2
cg16394157	0.732	0.739	0.751	0.008	15	HERC2
cg00796424	0.248	0.272	0.280	0.014	12	HOXC11
cg03762366	0.440	0.457	0.472	0.007	12	HOXC11
cg08962452	0.147	0.155	0.162	0.004	12	HOXC11
cg26386624	0.151	0.162	0.175	0.003	12	HOXC11
cg07123069	0.111	0.115	0.118	0.012	12	HOXC11
cg03536474	0.090	0.101	0.116	0.000	12	HOXC11
cg17273416	0.120	0.126	0.132	0.002	12	HOXC11
cg07797397	0.116	0.122	0.127	0.010	2	HOXD1
cg03450948	0.159	0.170	0.175	0.046	2	HOXD1
cg23420260	0.131	0.145	0.151	0.029	2	HOXD1
cg19542816	0.106	0.115	0.116	0.043	2	HOXD1
cg06697536	0.195	0.203	0.206	0.050	2	HOXD1
cg02746725	0.031	0.032	0.035	0.044	2	HOXD1
cg02466815	0.068	0.069	0.075	0.038	2	HOXD1
cg02527112	0.059	0.062	0.063	0.041	2	HOXD11
cg05942128	0.121	0.125	0.132	0.028	2	HOXD11

cg00495775	0.141	0.147	0.152	0.049	2	HOXD11
cg05500840	0.131	0.144	0.157	0.003	2	HOXD11
cg16632715	0.177	0.184	0.188	0.027	2	HOXD11
cg24633978	0.113	0.126	0.130	0.029	2	HOXD11
cg09406960	0.058	0.059	0.065	0.035	2	HOXD11
cg01708273	0.292	0.305	0.316	0.001	2	HOXD11
cg00035316	0.171	0.181	0.187	0.002	2	HOXD8
cg17863912	0.089	0.094	0.098	0.007	2	HOXD8
cg19384289	0.246	0.268	0.280	0.016	2	HOXD8
cg11969556	0.069	0.076	0.082	0.007	2	HOXD8
cg15808943	0.055	0.058	0.063	0.038	2	HOXD8
cg11261698	0.063	0.072	0.076	0.038	2	HOXD8
cg10239098	0.049	0.052	0.055	0.002	2	HOXD8
cg21351028	0.864	0.871	0.875	0.021	10	HPSE2
cg23852964	0.809	0.816	0.829	0.019	10	HPSE2
cg09902250	0.352	0.364	0.370	0.047	10	HPSE2
cg02800607	0.123	0.126	0.141	0.004	10	HPSE2
cg22823644	0.117	0.123	0.126	0.031	10	HPSE2
cg18499443	0.121	0.126	0.131	0.012	10	HPSE2
cg08927006	0.107	0.118	0.127	0.001	10	HPSE2
cg09511126	0.052	0.054	0.057	0.033	10	HPSE2
cg24936273	0.729	0.734	0.742	0.045	6	KHDRBS2
cg00472801	0.213	0.223	0.238	0.033	6	KHDRBS2
cg18239753	0.098	0.103	0.111	0.046	6	KHDRBS2
cg16587616	0.161	0.180	0.189	0.033	6	KHDRBS2
cg26715952	0.075	0.083	0.091	0.031	6	KHDRBS2
cg22014661	0.071	0.079	0.090	0.037	6	KHDRBS2
cg11315153	0.062	0.066	0.069	0.011	6	KHDRBS2
cg08189155	0.742	0.745	0.760	0.011	12	KSR2
cg08413096	0.639	0.648	0.668	0.013	12	KSR2
cg16471585	0.737	0.745	0.757	0.029	12	KSR2
cg21539988	0.850	0.851	0.856	0.034	12	KSR2
cg09293737	0.208	0.216	0.219	0.049	12	KSR2
cg26365508	0.032	0.032	0.036	0.017	12	KSR2
cg08271494	0.018	0.020	0.021	0.012	12	KSR2
cg01622379	0.125	0.130	0.132	0.042	1	LHX8
cg01185626	0.087	0.090	0.091	0.028	1	LHX8
cg22694818	0.240	0.255	0.266	0.002	1	LHX8
cg07563793	0.096	0.100	0.103	0.033	1	LHX8
cg23845450	0.216	0.221	0.236	0.023	1	LHX8
cg23952663	0.037	0.039	0.045	0.030	1	LHX8
cg19764599	0.040	0.049	0.052	0.000	1	LHX8
cg15343302	0.110	0.115	0.119	0.001	1	LHX8
cg08146483	0.110	0.124	0.126	0.038	1	LHX8

cg12764034	0.202	0.215	0.219	0.014	1	LHX8
cg00145253	0.196	0.202	0.204	0.037	1	LHX8
cg14245804	0.223	0.234	0.247	0.029	11	LRP5
cg11223669	0.731	0.735	0.746	0.004	11	LRP5
cg19986528	0.851	0.853	0.864	0.013	11	LRP5
cg12289926	0.693	0.698	0.714	0.044	11	LRP5
cg16192052	0.692	0.703	0.712	0.018	11	LRP5
cg01657329	0.770	0.772	0.783	0.007	11	LRP5
cg26525861	0.769	0.769	0.776	0.032	11	LRP5
cg02238051	0.865	0.868	0.875	0.009	16	MAPK8IP3
cg26989323	0.738	0.748	0.751	0.037	16	MAPK8IP3
cg08883140	0.624	0.633	0.636	0.043	16	MAPK8IP3
cg07780164	0.716	0.723	0.732	0.024	16	MAPK8IP3
cg26606899	0.980	0.981	0.982	0.032	16	MAPK8IP3
cg12384802	0.674	0.685	0.688	0.028	16	MAPK8IP3
cg06646646	0.900	0.904	0.905	0.024	16	MAPK8IP3
cg06286401	0.758	0.765	0.767	0.044	16	MAPK8IP3
cg05001715	0.057	0.062	0.067	0.031	5	MIR2277
cg25771615	0.047	0.050	0.055	0.006	5	MIR2277
cg19875969	0.101	0.112	0.121	0.035	5	MIR2277
cg22703659	0.037	0.045	0.053	0.001	5	MIR2277
cg17533458	0.076	0.092	0.099	0.033	5	MIR2277
cg07803236	0.085	0.100	0.102	0.047	5	MIR2277
cg06401019	0.084	0.092	0.097	0.002	5	MIR2277
cg03220633	0.070	0.074	0.077	0.021	5	MIR2277
cg12387713	0.056	0.060	0.065	0.047	5	MSX2
cg16328342	0.067	0.073	0.078	0.037	5	MSX2
cg15123984	0.157	0.168	0.184	0.017	5	MSX2
cg27096144	0.097	0.103	0.107	0.038	5	MSX2
cg20563910	0.109	0.123	0.133	0.013	5	MSX2
cg25323112	0.063	0.065	0.066	0.030	5	MSX2
cg06013117	0.281	0.299	0.301	0.020	5	MSX2
cg02071162	0.073	0.075	0.081	0.037	8	NKAIN3
cg01509237	0.060	0.061	0.062	0.048	8	NKAIN3
cg21498772	0.164	0.173	0.175	0.043	8	NKAIN3
cg08855288	0.159	0.174	0.177	0.038	8	NKAIN3
cg15858239	0.165	0.189	0.201	0.018	8	NKAIN3
cg11231949	0.235	0.260	0.279	0.044	8	NKAIN3
cg06410746	0.130	0.139	0.145	0.027	8	NKAIN3
cg21248274	0.160	0.164	0.171	0.037	8	NKAIN3
cg21191241	0.627	0.637	0.648	0.038	8	NKAIN3
cg22541831	0.383	0.389	0.407	0.019	8	NKAIN3
cg05904135	0.108	0.126	0.130	0.037	10	NKX6-2
cg16949120	0.115	0.131	0.142	0.003	10	NKX6-2

cg11163901	0.039	0.043	0.047	0.020	10	NKX6-2
cg01384488	0.112	0.123	0.133	0.005	10	NKX6-2
cg00626110	0.055	0.058	0.063	0.028	10	NKX6-2
cg10171448	0.154	0.164	0.169	0.016	10	NKX6-2
cg10182697	0.197	0.202	0.221	0.008	10	NKX6-2
cg00018024	0.027	0.028	0.033	0.016	10	NKX6-2
cg11428724	0.106	0.110	0.123	0.012	1	PAX7
cg08126203	0.075	0.078	0.084	0.006	1	PAX7
cg07221967	0.050	0.054	0.057	0.006	1	PAX7
cg22157239	0.085	0.089	0.093	0.043	1	PAX7
cg21215218	0.574	0.593	0.596	0.044	1	PAX7
cg15082028	0.049	0.054	0.063	0.001	1	PAX7
cg12751456	0.737	0.751	0.754	0.027	1	PAX7
cg05394124	0.637	0.652	0.653	0.012	1	PAX7
cg02114924	0.036	0.040	0.040	0.044	4	PCDH10
cg07665387	0.125	0.132	0.138	0.013	4	PCDH10
cg05401965	0.092	0.095	0.098	0.028	4	PCDH10
cg27600205	0.056	0.062	0.067	0.015	4	PCDH10
cg01408654	0.046	0.048	0.050	0.036	4	PCDH10
cg14410319	0.073	0.076	0.078	0.014	4	PCDH10
cg17504999	0.071	0.083	0.088	0.018	4	PCDH10
cg06667761	0.151	0.156	0.161	0.037	4	PCDH10
cg08172445	0.030	0.031	0.034	0.035	7	PEG10
cg21088896	0.044	0.046	0.049	0.030	7	PEG10
cg19924104	0.446	0.455	0.461	0.022	7	PEG10
cg11589966	0.489	0.495	0.505	0.031	7	PEG10
cg27435646	0.539	0.545	0.555	0.032	7	PEG10
cg22820921	0.383	0.389	0.400	0.047	7	PEG10
cg07278332	0.925	0.925	0.930	0.039	7	PEG10
cg21043213	0.333	0.361	0.369	0.008	4	PF4
cg16072462	0.364	0.393	0.399	0.014	4	PF4
cg15398841	0.401	0.433	0.437	0.010	4	PF4
cg02530824	0.322	0.352	0.359	0.015	4	PF4
cg06834998	0.380	0.417	0.427	0.006	4	PF4
cg05509609	0.397	0.431	0.447	0.014	4	PF4
cg13126871	0.354	0.376	0.380	0.029	4	PF4
cg15020983	0.796	0.798	0.813	0.022	4	PF4
cg25946758	0.095	0.097	0.100	0.014	10	PHYHIPL
cg11206067	0.066	0.071	0.076	0.040	10	PHYHIPL
cg01470088	0.154	0.165	0.177	0.019	10	PHYHIPL
cg26014391	0.084	0.094	0.097	0.013	10	PHYHIPL
cg13686115	0.074	0.075	0.078	0.037	10	PHYHIPL
cg23712342	0.157	0.169	0.186	0.008	10	PHYHIPL
cg08688023	0.224	0.239	0.256	0.006	10	PHYHIPL

cg03144922	0.252	0.262	0.272	0.005	4	PITX2
cg17507671	0.051	0.054	0.055	0.019	4	PITX2
cg05835105	0.216	0.239	0.246	0.008	4	PITX2
cg08867413	0.066	0.069	0.073	0.015	4	PITX2
cg05918492	0.170	0.175	0.182	0.034	4	PITX2
cg02725370	0.091	0.099	0.101	0.002	4	PITX2
cg26708319	0.143	0.156	0.167	0.000	4	PITX2
cg23663774	0.083	0.087	0.091	0.025	4	PITX2
cg10391633	0.365	0.372	0.379	0.042	4	PITX2
cg06842954	0.141	0.149	0.155	0.011	4	PITX2
cg19597382	0.099	0.106	0.112	0.035	4	POU4F2
cg13262687	0.083	0.085	0.088	0.032	4	POU4F2
cg24199834	0.122	0.129	0.141	0.006	4	POU4F2
cg25406138	0.094	0.096	0.100	0.034	4	POU4F2
cg06083330	0.157	0.169	0.180	0.003	4	POU4F2
cg00505045	0.148	0.153	0.154	0.035	4	POU4F2
cg16887264	0.064	0.069	0.079	0.021	4	POU4F2
cg11801490	0.755	0.766	0.768	0.028	14	PPP2R5E
cg03161847	0.828	0.831	0.834	0.039	14	PPP2R5E
cg02645147	0.697	0.702	0.727	0.010	14	PPP2R5E
cg14703403	0.673	0.686	0.708	0.001	14	PPP2R5E
cg04613771	0.719	0.729	0.746	0.006	14	PPP2R5E
cg03875433	0.029	0.030	0.032	0.004	14	PPP2R5E
cg05844852	0.022	0.025	0.025	0.041	14	PPP2R5E
cg06604497	0.910	0.914	0.918	0.038	12	PRH1
cg17715732	0.855	0.856	0.866	0.039	12	PRH1
cg18241189	0.498	0.503	0.534	0.005	12	PRH1
cg05750898	0.698	0.708	0.720	0.044	12	PRH1
cg18371539	0.582	0.586	0.601	0.022	12	PRH1
cg13217184	0.847	0.852	0.863	0.001	12	PRH1
cg08555389	0.770	0.778	0.791	0.032	12	PRH1
cg20418818	0.491	0.501	0.513	0.025	12	PRH1
cg18421318	0.728	0.735	0.761	0.013	12	PRH1
cg13942242	0.649	0.657	0.665	0.023	12	PRH1
cg21216543	0.038	0.040	0.040	0.032	12	PRH1
cg12647643	0.042	0.044	0.046	0.020	4	PTPN13
cg04397429	0.043	0.045	0.047	0.004	4	PTPN13
cg15543566	0.114	0.129	0.136	0.013	4	PTPN13
cg13781843	0.059	0.069	0.071	0.032	4	PTPN13
cg21408445	0.688	0.701	0.712	0.020	4	PTPN13
cg02874994	0.679	0.693	0.703	0.034	4	PTPN13
cg19757586	0.764	0.772	0.783	0.039	4	PTPN13
cg14615491	0.383	0.393	0.418	0.019	7	RBM33
cg22551062	0.648	0.687	0.742	0.000	7	RBM33

cg16595042	0.802	0.807	0.813	0.042	7	RBM33
cg10541993	0.388	0.392	0.420	0.034	7	RBM33
cg03088741	0.465	0.473	0.485	0.022	7	RBM33
cg22172340	0.691	0.703	0.726	0.043	7	RBM33
cg04278580	0.713	0.723	0.728	0.031	7	RBM33
cg04774597	0.242	0.222	0.202	0.014	15	RPLP1
cg26218577	0.355	0.330	0.304	0.009	15	RPLP1
cg07513768	0.176	0.158	0.148	0.010	15	RPLP1
cg00469015	0.170	0.150	0.128	0.002	15	RPLP1
cg11437810	0.178	0.157	0.140	0.042	15	RPLP1
cg10716823	0.129	0.116	0.103	0.006	15	RPLP1
cg11945251	0.041	0.040	0.038	0.033	15	RPLP1
cg14070647	0.060	0.063	0.065	0.008	8	RSPO2
cg20061155	0.072	0.074	0.081	0.038	8	RSPO2
cg04549460	0.076	0.081	0.087	0.003	8	RSPO2
cg22600043	0.203	0.210	0.222	0.006	8	RSPO2
cg16845394	0.125	0.130	0.139	0.023	8	RSPO2
cg13700897	0.095	0.098	0.104	0.048	8	RSPO2
cg00997551	0.242	0.252	0.269	0.006	8	RSPO2
cg17945440	0.472	0.489	0.502	0.028	8	RSPO2
cg14063488	0.053	0.056	0.058	0.023	4	SFRP2
cg03202804	0.065	0.068	0.072	0.021	4	SFRP2
cg23207990	0.146	0.160	0.174	0.001	4	SFRP2
cg07999845	0.108	0.111	0.119	0.007	4	SFRP2
cg24241928	0.202	0.207	0.218	0.001	4	SFRP2
cg01298731	0.524	0.532	0.549	0.031	4	SFRP2
cg05241277	0.271	0.272	0.287	0.014	4	SFRP2
cg01566785	0.552	0.557	0.568	0.026	7	SGCE
cg13660372	0.527	0.536	0.558	0.002	7	SGCE
cg27230044	0.387	0.397	0.400	0.014	7	SGCE
cg26997085	0.346	0.354	0.361	0.028	7	SGCE
cg22331138	0.443	0.445	0.451	0.047	7	SGCE
cg27120649	0.433	0.447	0.457	0.023	7	SGCE
cg27001184	0.454	0.460	0.465	0.030	7	SGCE
cg11175683	0.425	0.432	0.440	0.034	7	SGCE
cg06695761	0.434	0.438	0.448	0.029	7	SGCE
cg10536264	0.774	0.779	0.788	0.010	5	SHROOM1
cg21811204	0.396	0.429	0.440	0.000	5	SHROOM1
cg13218710	0.183	0.193	0.201	0.008	5	SHROOM1
cg24789467	0.388	0.407	0.414	0.003	5	SHROOM1
cg16609957	0.179	0.185	0.191	0.011	5	SHROOM1
cg15162922	0.793	0.797	0.804	0.021	5	SHROOM1
cg13662483	0.791	0.795	0.800	0.024	5	SHROOM1
cg00605982	0.093	0.096	0.099	0.035	3	SLC6A11

cg07744841	0.191	0.208	0.221	0.000	3	SLC6A11
cg22331862	0.350	0.360	0.377	0.025	3	SLC6A11
cg21123160	0.326	0.343	0.374	0.000	3	SLC6A11
cg16435571	0.074	0.079	0.086	0.017	3	SLC6A11
cg09660365	0.304	0.316	0.322	0.034	3	SLC6A11
cg24419134	0.839	0.845	0.847	0.028	3	SLC6A11
cg25194918	0.100	0.103	0.107	0.032	2	SOX11
cg04225243	0.086	0.089	0.094	0.021	2	SOX11
cg08432727	0.095	0.100	0.105	0.021	2	SOX11
cg08165221	0.088	0.094	0.096	0.003	2	SOX11
cg20008332	0.062	0.070	0.080	0.001	2	SOX11
cg18863333	0.139	0.145	0.156	0.019	2	SOX11
cg24837370	0.149	0.155	0.161	0.050	2	SOX11
cg15989068	0.161	0.171	0.182	0.024	2	SOX11
cg04868167	0.014	0.015	0.017	0.023	4	SPATA18
cg26039254	0.020	0.023	0.028	0.003	4	SPATA18
cg06341054	0.046	0.050	0.054	0.000	4	SPATA18
cg10418567	0.041	0.043	0.044	0.034	4	SPATA18
cg09022993	0.079	0.083	0.087	0.014	4	SPATA18
cg01429039	0.071	0.076	0.082	0.000	4	SPATA18
cg05717382	0.064	0.070	0.075	0.000	4	SPATA18
cg24395452	0.226	0.235	0.243	0.018	4	SPATA18
cg02074501	0.654	0.667	0.690	0.040	2	SPTBN1
cg14313048	0.796	0.806	0.811	0.024	2	SPTBN1
cg01936472	0.206	0.220	0.244	0.010	2	SPTBN1
cg16152092	0.087	0.089	0.110	0.008	2	SPTBN1
cg11288670	0.091	0.093	0.100	0.021	2	SPTBN1
cg01766943	0.615	0.618	0.644	0.040	2	SPTBN1
cg24528602	0.828	0.832	0.838	0.044	2	SPTBN1
cg21270675	0.857	0.859	0.872	0.002	2	SPTBN1
cg06306219	0.079	0.080	0.083	0.036	4	TRIM2
cg25922624	0.312	0.320	0.330	0.020	4	TRIM2
cg25173574	0.819	0.824	0.828	0.030	4	TRIM2
cg12453905	0.083	0.086	0.096	0.021	4	TRIM2
cg02559915	0.779	0.785	0.787	0.025	4	TRIM2
cg19717235	0.019	0.020	0.022	0.014	4	TRIM2
cg20347377	0.506	0.521	0.522	0.031	4	TRIM2
cg22546302	0.618	0.621	0.657	0.037	4	TRIM2
cg04043591	0.047	0.050	0.052	0.013	7	TWIST1
cg20052718	0.069	0.076	0.084	0.025	7	TWIST1
cg17839237	0.048	0.056	0.059	0.006	7	TWIST1
cg14391419	0.050	0.054	0.056	0.048	7	TWIST1
cg10126205	0.071	0.076	0.085	0.009	7	TWIST1
cg21424940	0.087	0.090	0.096	0.030	7	TWIST1

cg10624122	0.048	0.050	0.052	0.017	7	TWIST1
cg02109793	0.173	0.181	0.188	0.004	22	UPB1
cg03364108	0.214	0.234	0.252	0.003	22	UPB1
cg15552843	0.280	0.296	0.311	0.010	22	UPB1
cg03503516	0.342	0.357	0.381	0.002	22	UPB1
cg12492885	0.405	0.422	0.439	0.005	22	UPB1
cg02469909	0.328	0.347	0.363	0.003	22	UPB1
cg12550055	0.234	0.242	0.248	0.025	22	UPB1
cg18495047	0.738	0.759	0.765	0.021	22	UPB1
cg11984989	0.137	0.145	0.150	0.044	7	WDR60
cg18734591	0.808	0.816	0.822	0.004	7	WDR60
cg04463719	0.955	0.957	0.963	0.003	7	WDR60
cg26321613	0.711	0.722	0.726	0.020	7	WDR60
cg24397884	0.553	0.572	0.604	0.018	7	WDR60
cg16704129	0.855	0.858	0.863	0.032	7	WDR60
cg02422616	0.688	0.695	0.701	0.041	7	WDR60
cg24306648	0.952	0.956	0.963	0.041	1	WDR8
cg19270286	0.805	0.806	0.820	0.035	1	WDR8
cg04021697	0.114	0.124	0.142	0.021	1	WDR8
cg21000072	0.096	0.101	0.105	0.044	1	WDR8
cg16823083	0.381	0.407	0.423	0.008	1	WDR8
cg24678611	0.180	0.189	0.197	0.003	1	WDR8
cg12475507	0.250	0.258	0.267	0.014	1	WDR8
cg24073122	0.244	0.254	0.261	0.025	1	WDR8
cg04201521	0.044	0.046	0.050	0.050	2	WNT6
cg19837003	0.689	0.693	0.703	0.007	2	WNT6
cg05456561	0.740	0.750	0.765	0.006	2	WNT6
cg05618201	0.810	0.810	0.817	0.033	2	WNT6
cg06795233	0.316	0.322	0.346	0.019	2	WNT6
cg22587479	0.114	0.125	0.148	0.016	2	WNT6
cg00011225	0.100	0.108	0.131	0.030	2	WNT6
cg14639163	0.199	0.209	0.220	0.022	2	WNT6
cg14649945	0.764	0.771	0.774	0.017	11	WT1
cg25094569	0.069	0.074	0.077	0.014	11	WT1
cg04456238	0.338	0.351	0.356	0.018	11	WT1
cg15518358	0.398	0.409	0.428	0.021	11	WT1
cg26848718	0.099	0.103	0.108	0.041	11	WT1
cg14657517	0.048	0.052	0.057	0.009	11	WT1
cg07565441	0.071	0.078	0.079	0.045	11	WT1
cg20134916	0.044	0.045	0.052	0.024	11	WT1
cg26232818	0.056	0.057	0.061	0.042	11	WT1
cg05768078	0.761	0.772	0.777	0.017	16	WWP2
cg07265339	0.827	0.832	0.836	0.022	16	WWP2
cg27456203	0.784	0.792	0.795	0.046	16	WWP2

cg02147637	0.867	0.871	0.873	0.039	16	WWP2
cg03417340	0.759	0.762	0.769	0.029	16	WWP2
cg07281938	0.822	0.825	0.830	0.034	16	WWP2
cg04633966	0.747	0.750	0.760	0.013	16	WWP2
cg09719477	0.014	0.016	0.016	0.037	11	ZBTB16
cg16384885	0.024	0.025	0.028	0.046	11	ZBTB16
cg09360036	0.423	0.427	0.433	0.047	11	ZBTB16
cg06493289	0.730	0.735	0.742	0.028	11	ZBTB16
cg09994445	0.683	0.689	0.710	0.002	11	ZBTB16
cg10827488	0.139	0.146	0.151	0.014	11	ZBTB16
cg22130673	0.653	0.658	0.670	0.019	11	ZBTB16
cg14786860	0.784	0.791	0.795	0.011	11	ZBTB16
cg10397223	0.745	0.750	0.756	0.029	11	ZBTB16
cg08047455	0.602	0.606	0.618	0.013	11	ZBTB16
cg14289985	0.097	0.103	0.115	0.009	19	ZNF471
cg14042851	0.091	0.100	0.112	0.005	19	ZNF471
cg11539780	0.083	0.098	0.112	0.002	19	ZNF471
cg19811761	0.154	0.178	0.199	0.002	19	ZNF471
cg12844912	0.106	0.124	0.132	0.037	19	ZNF471
cg00674365	0.186	0.214	0.238	0.009	19	ZNF471
cg19358877	0.093	0.108	0.117	0.012	19	ZNF471
cg24713204	0.226	0.238	0.246	0.016	19	ZNF471
cg02823803	0.189	0.195	0.202	0.031	19	ZNF471
cg25927516	0.845	0.850	0.855	0.024	19	ZNF577
cg06878361	0.265	0.277	0.281	0.001	19	ZNF577
cg03562414	0.292	0.308	0.313	0.039	19	ZNF577
cg10635122	0.222	0.236	0.245	0.007	19	ZNF577
cg24794228	0.411	0.433	0.441	0.047	19	ZNF577
cg10783469	0.321	0.345	0.358	0.005	19	ZNF577
cg23010048	0.277	0.292	0.298	0.008	19	ZNF577
cg11269599	0.239	0.252	0.257	0.033	19	ZNF577
cg22331349	0.354	0.368	0.369	0.016	19	ZNF577
cg12227172	0.217	0.232	0.236	0.018	19	ZNF577
cg01483656	0.203	0.243	0.266	0.016	19	ZNF714
cg19882830	0.290	0.346	0.389	0.011	19	ZNF714
cg17751872	0.331	0.362	0.380	0.005	19	ZNF714
cg01326874	0.332	0.389	0.430	0.005	19	ZNF714
cg15174294	0.204	0.234	0.254	0.006	19	ZNF714
cg06238316	0.231	0.267	0.295	0.002	19	ZNF714
cg09352518	0.271	0.316	0.355	0.004	19	ZNF714
cg08333627	0.620	0.634	0.652	0.022	19	ZNF714
cg16896134	0.734	0.738	0.759	0.046	19	ZNF714
cg10186229	0.771	0.782	0.802	0.013	19	ZNF83
cg18611631	0.741	0.750	0.759	0.033	19	ZNF83

cg03228288	0.047	0.050	0.065	0.014	19	ZNF83
cg15008743	0.142	0.147	0.183	0.012	19	ZNF83
cg25793430	0.457	0.470	0.503	0.000	19	ZNF83
cg15209676	0.498	0.525	0.548	0.001	19	ZNF83
cg08318732	0.400	0.414	0.424	0.006	19	ZNF83
cg20875196	0.045	0.047	0.048	0.009	19	ZNF83
cg21331821	0.058	0.064	0.074	0.003	19	ZSCAN1
cg24368848	0.257	0.280	0.298	0.011	19	ZSCAN1
cg25537993	0.301	0.319	0.336	0.022	19	ZSCAN1
cg19819285	0.104	0.113	0.121	0.006	19	ZSCAN1
cg10132208	0.259	0.272	0.290	0.025	19	ZSCAN1
cg20449685	0.203	0.214	0.232	0.026	19	ZSCAN1
cg05983315	0.140	0.149	0.157	0.036	19	ZSCAN1
cg27497487	0.746	0.747	0.761	0.014	19	ZSCAN1
